# Association of mitochondrial haplogroup H with reduced risk of type 2 Diabetes among Gulf Region Arabs

**DOI:** 10.3389/fendo.2024.1443737

**Published:** 2024-11-26

**Authors:** Mohammed Dashti, Naser M. Ali, Hussain Alsaleh, Sumi Elsa John, Rasheeba Nizam, Thangavel Alphonse Thanaraj, Fahd Al-Mulla

**Affiliations:** ^1^ Genetics and Bioinformatics Department, Dasman Diabetes Institute, Kuwait City, Kuwait; ^2^ Department of Medical Laboratories, Ahmadi Hospital, Kuwait Oil Company (KOC), Ahmadi, Kuwait; ^3^ Saad Al-Abdullah Academy for Security Sciences, Ministry of Interior, Shuwaikh, Kuwait

**Keywords:** mitochondria, type 2 diabetes, haplogroups, mtDNA variants, Arab

## Abstract

**Background:**

Numerous studies have linked mitochondrial dysfunction to the development of type 2 diabetes (T2D) by affecting glucose-stimulated insulin secretion in pancreatic beta cells and reducing oxidative phosphorylation in insulin-responsive tissues. Given the strong genetic underpinnings of T2D, research has explored the connection between mitochondrial DNA haplogroups, specific variants, and the risk and comorbidities of T2D. For example, haplogroups F, D, M9, and N9a have been linked to an elevated risk of T2D across various populations. Additionally, specific mitochondrial DNA variants, such as the rare mtDNA 3243 A>G and the more prevalent mtDNA 16189 T>C, have also been implicated in heightened T2D risk. Notably, these associations vary among different populations. Given the high incidence of T2D in the Gulf Cooperation Council countries, this study investigates the correlation between T2D and mitochondrial haplogroups and variants in Arab populations from the Gulf region.

**Methods:**

This analysis involved mitochondrial haplogroup and variant testing in a cohort of 1,112 native Kuwaiti and Qatari individuals, comprising 685 T2D patients and 427 controls. Complete mitochondrial genomes were derived from whole exome sequencing data to examine the associations between T2D and haplogroups and mitochondrial DNA variants.

**Results:**

The analysis revealed a significant protective effect of haplogroup H against T2D (odds ratio [OR] = 0.65; P = 0.022). This protective association persisted when adjusted for age, sex, body mass index (BMI) and population group, with an OR of 0.607 (P = 0.021). Furthermore, specific mitochondrial variants showed significant associations with T2D risk after adjustment for relevant covariates, and some variants were exclusively found in T2D patients.

**Conclusion:**

Our findings confirm that the maternal haplogroup H, previously identified as protective against obesity in Kuwaiti Arabs, also serves as a protective factor against T2D in Arabs from the Gulf region. The study also identifies mitochondrial DNA variants that either increase or decrease the risk of T2D, underscoring their role in cellular energy metabolism.

## Introduction

Type 2 diabetes (T2D) is a chronic adult-onset metabolic disorder characterized by high blood sugar due to insulin resistance or inadequate insulin production by pancreatic beta cells. Major complications of T2D include retinopathy, nephropathy, and neuropathy, which can lead to blindness, renal failure, impotence, and foot amputation, respectively (1). The International Diabetes Federation (IDF) has reported that 73 million people have diabetes in the Middle East and North Africa region, with this number projected to increase to 95 million by 2030. Kuwait has a high prevalence of 25.5%, and Qatar has a prevalence of 16.4% among the total adult population (2).

T2D is known to have a heritable component, yet the interactions between environmental and genetic factors complicate their identification. The currently known susceptibility genes for T2D, identified through linkage analysis, candidate gene approaches, and genome-wide association studies on the nuclear genome, contribute only a small percentage to the high estimated genetic heritability of T2D (3–5), suggesting the presence of missing heritability (6).

Mitochondria play a critical role in generating cellular energy, primarily through the process of oxidative phosphorylation (OXPHOS), where adenosine triphosphate (ATP) is synthesized within the inner mitochondrial membrane. Beyond energy production, mitochondria are key players in fatty acid metabolism and the production of reactive oxygen species (ROS), which are essential for oxidative stress responses and the regulation of apoptosis (7). Mitochondrial dysfunction, including reduced oxidative phosphorylation capacity, has been linked to insulin resistance and T2D in classic target organs such as the liver, fat, and muscle (8–11).

The mitochondrial genome (mtDNA) is a maternally inherited, circular, double-stranded molecule that is independent of nuclear DNA. Each cell contains several thousand mitochondria, each housing multiple copies of mtDNA (12, 13). A notable feature of the mtDNA is its proneness to high rates of genetic alteration compared to nuclear DNA especially the D-loop region which is a major non-coding region that oversees mitochondrial transcription and replication (14). Haplogroups, which group individuals based on similar mtDNA variants, provide insight into genetic diversity and evolutionary history across different populations. These variations, including single-nucleotide polymorphisms (SNPs) and insertions or deletions (INDELs), are increasingly recognized as potential markers for diagnosing and understanding complex common disorders (15, 16). Some mtDNA mutations are heteroplasmic, leading to variability in phenotypic expression because the proportion of mtDNA copies with the pathogenic variant is higher than that of wild-type mtDNA copies (17).

There are mitochondrial DNA mutations associated with T2D exhibiting population-wide variation in allele frequencies, for instance, a rare mutation, mtDNA 3243 A>G in the tRNA(Leu)(UUR) gene, causes maternally inherited diabetes and deafness (MIDD); however, its frequency varies across different ethnicities (18, 19). Another example is a common variant, mtDNA 16189 T>C which is associated with an increased risk of T2D in populations with a high frequency of this variant, such as Asian populations (20), but not in European (21) and North African populations (22), where the frequency is low (23). Additionally, the mtDNA haplogroup is proposed to be involved in risk prediction of T2D. Haplogroups F, D, and M9 are associated with an increased risk of T2D (21, 24, 25). However, these haplogroup associations are not consistent across populations; for example, haplogroup N9a is associated with an increased risk of T2D in southern Chinese populations (26), whereas haplogroup N9a is associated with protection against T2D in Japanese and Korean populations (24).

Despite extensive research on mitochondrial dysfunction and diabetes in other regions, there is a significant gap in studies focused on the Arab Gulf region. Studying mitochondrial variants and haplogroups in this region is crucial due to the high prevalence of type 2 diabetes and the unique genetic makeup of these populations. The primary objective of this study is to fill this gap by analysing mtDNA variants and haplogroups in Kuwait and Qatar to understand their impact on diabetes susceptibility. By way of utilizing whole mitochondrial genome sequences extracted from whole exome data of 1,112 individuals from Kuwait and Qatar, this study aims to clarify the relationship of mtDNA haplogroups/variants and type 2 diabetes, potentially enhancing precision medicine by improving the identification of individuals at risk based on their genetic profiles.

## Materials and methods

### Ethics declaration

This study received approval from the Institutional Ethical Review Committee at Dasman Diabetes Institute, following the ethical principles outlined in the Declaration of Helsinki. While the Qatari whole-exome sequence data utilized in this research were previously published in a separate study by Fakhro et al. (27) and O’Beirne et al. (28), the study adhered to ethical standards, and written informed consent was obtained from all participants. The ethical approval for the study, including the use of the previously collected samples, was granted under the project reference number RA HM 2019-025.

### Whole exome data

This secondary analysis involved a cohort of 1,112 unrelated participants from Kuwait and Qatar, collected through national genome sequencing programs designed to investigate genomic variants at the population level. DNA from whole blood of 348 Kuwaiti individuals was sequenced using the Illumina HiSeq platform with TruSeq and Nextera capture kits, as previously described by John et al. (29) (SRA accession: https://www.ncbi.nlm.nih.gov/bioproject/PRJNA1162699). DNA from whole blood of 764 Qatari participants with available clinical data (**Supplementary Table S1**) was sequenced using the Illumina HiSeq platform with the SureSelect Agilent V5 kit, which captures the entire mitochondrial genome (27, 28) (SRA accession: https://www.ncbi.nlm.nih.gov/bioproject/?term=PRJNA290484).

Participants were diagnosed with Type 2 Diabetes based on the American Diabetes Association (ADA) criteria, including fasting blood glucose ≥126 mg/dL and/or HbA1C ≥6.5%. The study included individuals of Arab ethnicity with confirmed ancestry up to three generations. For detailed inclusion criteria, please refer to Hebbar et al. (30) for the Kuwaiti cohort and O’Beirne et al. (28) for the Qatari cohort.

### mtDNA sequences, variant calling, and annotation

The FASTQ paired-end Illumina reads were aligned to the human reference assembly GRCh37 with the default setting of Burrows-Wheeler Aligner (BWA-MEM) version v07-17 (31). After alignment, duplicate reads were removed, and the mtDNA sequence (NC_012920.1) was extracted utilizing Picard tool version 2.20.2 (http://broadinstitute.github.io/picard) and SAMtools version 0.1.19 (32). Subsequently, the mtDNA coverage and Genomic Variant Call Format (GVCF) files were generated for each sample with the Genome Analysis Tool Kit (GATK) version v3.8-1-0 (33). Using GATK HaploCaller, the combined 1,112 GVCF files were genotyped, producing a Variant Calling Format (VCF) file that contained the identified mtDNA variants. Finally, mtDNA annotations were performed using the Ensembl Variant Effect Predictor (VEP) (34), the Mitomap (https://www.mitomap.org/) database (35), and Varsome website (https://varsome.com).

### mtDNA haplogroup assignment

To determine the mitochondrial haplogroup profiles for all 1,112 individuals, the mtDNA VCF files were analysed using HaploGrep 2 (36) with the phylotree build PT17-FU1 (accessed on February 2, 2024).

### Statistical analyses

Statistical analyses were performed using R software version 3.6.2 (https://www.R-project.org/). Descriptive statistics were derived for demographic and anthropometric data, including age, BMI, and T2D status. Continuous variables were summarized as mean ± standard deviation (SD) or as median and interquartile range (IQR). The Chi-square test was used to assess the statistical significance of associations between categorical variables (sex and population) and T2D status. The Mann-Whitney U test was used to examine the associations between age, BMI, and T2D status. A p-value of less than 0.05 was considered statistically significant.

To identify hidden relationships among the samples due to potential covariates, principal component analysis (PCA) was conducted on the complete set of mtDNA variants. PCA was carried out using the PCAtools package in R, and the results were visualized on a biplot, highlighting the principal components (PCs) that captured the most variation in the data.

Fisher’s exact test was employed to test for nominal associations between T2D and mtDNA haplogroups. The odds ratio (OR) and 95% confidence intervals (CI) were calculated for each haplogroup, with a p-value <0.05 set as the threshold for statistical significance. Additionally, logistic regression was implemented using IBM^®^ SPSS^®^ Statistics Version 25 software to adjust for covariates, including sex, age, BMI, and population. Finally, PLINK version 1.9 (71) was used to identify mtDNA variants associated with T2D, with a two-tailed p-value <0.05. In addition, PLINK tool was used for conditional analysis on the top leading variant with the lowest p-value. Independence of associated variants was assessed using LD pruning with the parameters (–indep-pairwise 50 5 0).

## Results

### Study demographic and clinical profile

The samples in our study, including those referenced from previous research (27, 29), underwent relatedness assessments based on nuclear DNA to ensure unrelated samples. **Table 1** presents descriptive statistics for the dataset containing 1,112 Kuwaiti and Qatari individuals. The Mann-Whitney U test displayed significant differences between T2D and control individuals with regards to age categories and BMI scores. Additionally, the Chi-square test showed a significant result for the distribution of T2D and control individuals with regards to population (Kuwaiti and Qatari). However, no significant difference was found for sex distribution between the two groups.

**Table 1:** Demographic and anthropometric information of the study participants.

	Control	T2D	Total	P-value
Sex				
Male	175 (41.08%)	294 (42.92%)	469 (42.21%)	0.5
Female	251 (58.92%)	391 (57.08%)	642 (57.79%)	
Population				
Kuwaiti	165 (38.73%)	182 (26.57%)	347 (31.23%)	2.83E-05
Qatari	261 (61.27%)	503 (73.43%)	764 (68.77%)	
Age (years)				
≤ 50	313 (73.47%)	187 (27.30%)	500 (45%)	9.9E-51
> 50	113 (26.53%)	498 (72.70%)	611 (55%)	
Mean ± SD	44.43 ± 11.88	56.97 ± 10.47	52.16 ± 12.60	
Median (IQR)	43.0 (35.0-51.0)	57.0 (50-64)	52.0 (43-60.5)	
BMI				
Mean ± SD	31.89 ± 8.05	33.28 ± 6.90	32.74 ± 7.39	0.0007
Median (IQR)	31.5 (25.4-33.4)	32.5 (27.9-37.9)	31.6 (27.5-37.1)	

P-values for age categories and BMI for T2D vs. control were calculated using the Mann–Whitney U test. P-values for sex and population counts in T2D vs. control were calculated using the Chi-square test.

### mtDNA variants

A total of 1,850 high-quality variants were identified after passing filters and quality control steps. These identified mtDNA variants include SNPs and INDELs, with an average coverage of 75X across the entire mtDNA. The genomic-control inflation factor (λ) was 1 in tests that accounted for all the covariates. Since this value is close to 1.0, it indicates that there is no need to adjust the association statistics for genomic-control inflation.

### mtDNA haplogroups

**Figure 1:**
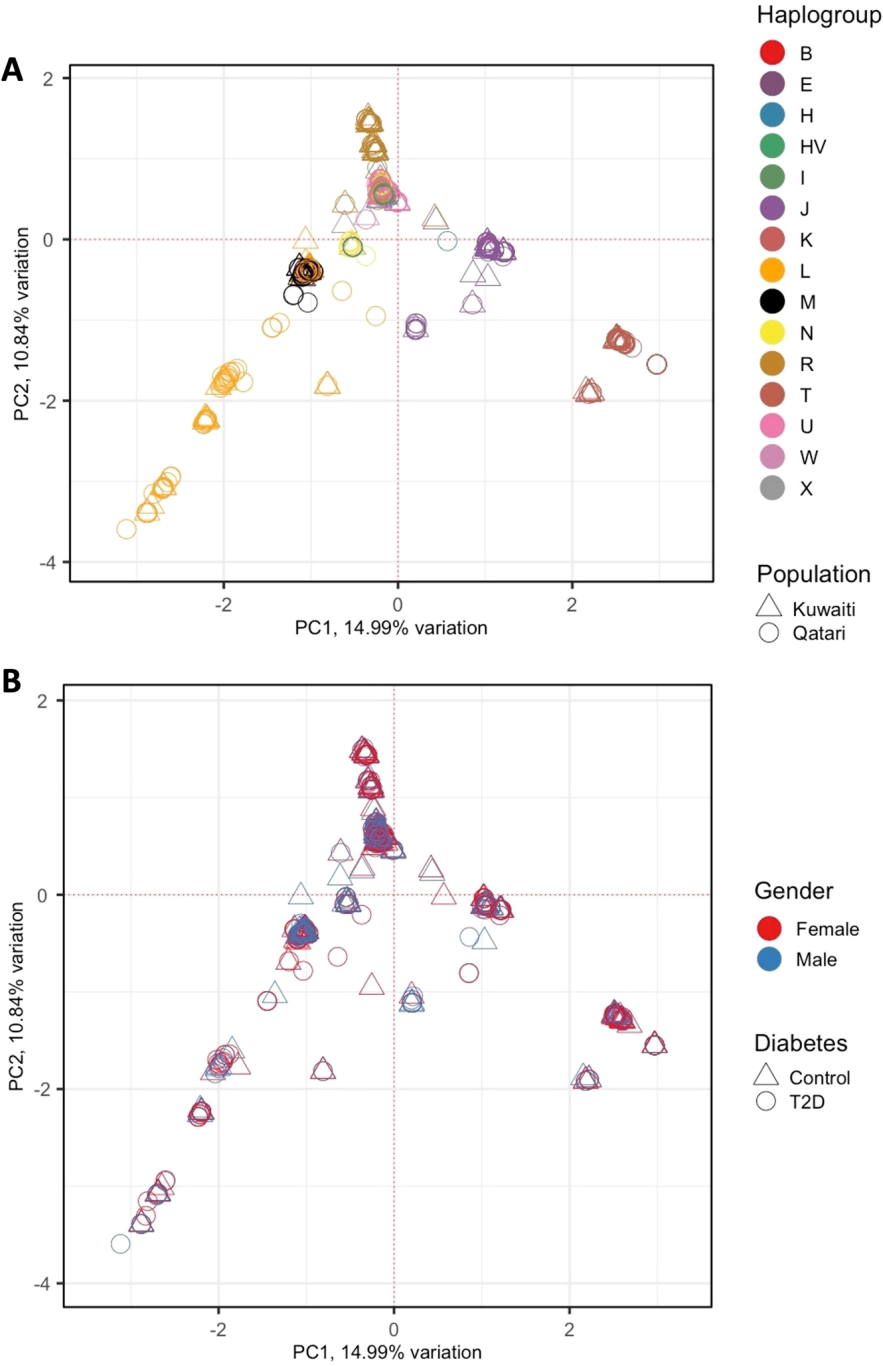
PCA of 1,112 Samples of Gulf Region Arabs Based on Their mtDNA Variants. The PCA was conducted on all mitochondrial DNA variants identified in the two cohorts. PC1 (Principal Component 1) and PC2 (Principal Component 2) on the x- and y-axes represent the first two principal components, which capture the largest proportion of genetic variance in the dataset. The percentage of variance explained by each component is indicated on the axes. **(A)** The samples are colour-coded based on their mitochondrial haplogroups, reflecting the rich ancestral information contained in the mtDNA, and different shapes describe the population groups. **(B)** The same PCA plot is overlaid with gender represented by different colours and diabetes status represented by different shapes.

The average haplogroup quality score was above 92%, obtained using HaploGrep 2.4.0, which identified 15 mitochondrial haplogroups (B, E, H, HV, I, J, K, L, M, N, R, T, U, W, X). PCA was performed on the mitochondrial DNA variants from both cohorts to explore potential hidden relationships among the samples. The PCA results revealed that the 1,112 samples clustered primarily according to their mitochondrial haplogroups (**Figure 1A**), reflecting the rich ancestral information encoded in the mtDNA. Notably, the samples did not cluster based on population groups (Kuwaiti or Qatari), indicating that the primary genetic variation is driven by haplogroup differences rather than geographical origin. In **Figure 1B**, the PCA plot shows no distinct clustering based on gender or diabetes status, further emphasizing that mitochondrial haplogroups are the dominant factor influencing the genetic structure observed in this analysis.

### mtDNA haplogroups associated with T2D

**Figure 2:**
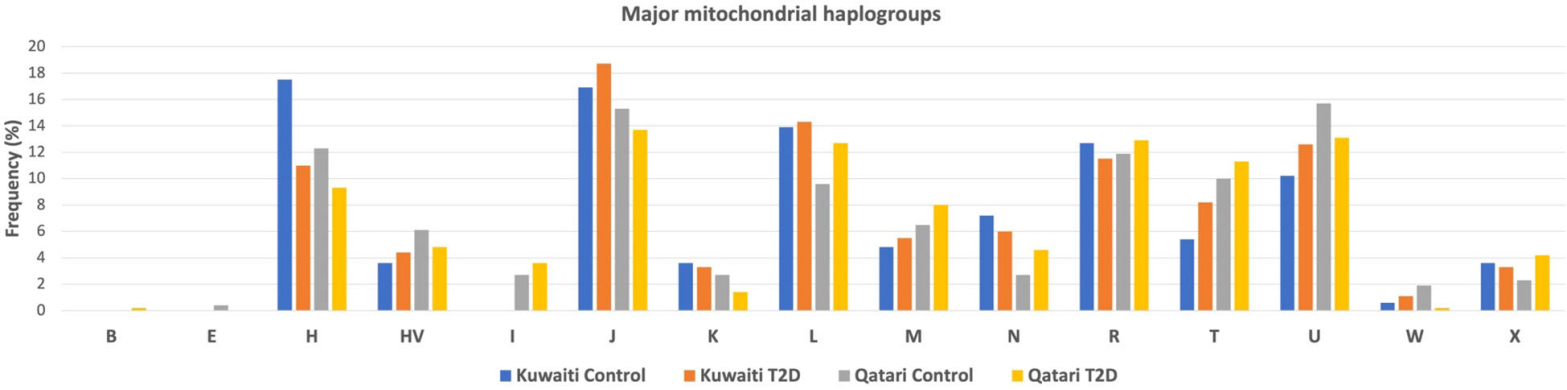
Distribution of mtDNA Haplogroups of gulf region arabs based on their diabetes status. Allocation of major mtDNA haplogroup prevalence in T2D and control groups within the Kuwaiti and Qatari populations.

**Figure 2** presents the frequencies of mitochondrial haplogroups in the Gulf region Arabs of Kuwaiti and Qatari population based on diabetes phenotype. The frequency of the H haplogroup was higher in the control groups compared to the diabetes groups in both the Kuwaiti (17.5% vs. 11%) and the Qatari (12.3% vs. 9.3%) cohorts (**Figure 2**). The most common haplogroups were J followed by U haplogroup. Haplogroups with frequencies below 3% were categorized as “others”.

When conducting separate mitochondrial haplogroup analysis using the Fisher exact test on the Kuwaiti population (**Supplementary Table S2**) and the Qatari population (**Supplementary Table S3**), we did not find significant associations. However, when we combined the two populations as Arabs in the Gulf region, thereby increasing the number of samples, we observed a significant association using the multivariate logistic regression analysis (**Table 2**). Specifically, the H haplogroup had a protective effect against obesity (odds ratio [OR] = 0.65; p = 0.022). This effect remained significant after adjusting for age, sex, BMI, and population (OR/95% CI = 0.607/0.397-0.929; p = 0.021). Further analysis showed that most individuals with the H haplogroup belonged to the H14b and H2a subclades (**Table 3**).

**Table 2:** mtDNA Haplogroups association of gulf region arabs for diabetes.

Haplogroup	Kuwait	Qatar	Total	T2D	Control	OR	*P*-value	OR (95%CI)* after covariates adjustment	P-value* after covariates adjustment
	N (348)	N (764)	N (1,112)	N (685)	N (427)				
H	49 (14.1%)	78 (10.2%)	127 (11.4%)	67 (9.8%)	61 (14.3%)	0.65	0.022	0.607 (0.397-0.929)	0.021
HV	14 (4%)	40 (5.2%)	54 (4.9%)	32 (4.7%)	22 (5.2%)	0.9	0.717	0.803 (0.427-1.511)	0.497
J	62 (17.8%)	109 (14.3%)	171 (15.4%)	103 (15%)	68 (15.9%)	0.93	0.690	0.928 (0.634-1.359)	0.702
L	49 (14.1%)	89 (11.6%)	138 (12.4%)	90 (13.1%)	48 (11.2%)	1.19	0.351	1.320 (0.861-2.024)	0.203
M	18 (5.2%)	57 (7.5%)	75 (6.7%)	50 (7.3%)	25 (5.8%)	1.27	0.350	1.148 (0.656-2.009)	0.629
N	23 (6.6%)	30 (3.8%)	53 (4.8%)	34 (5%)	19 (4.4%)	1.12	0.696	1.048 (0.547-2.008)	0.888
R	42 (12.1%)	96 (12.6%)	138 (12.4%)	86 (12.6%)	52 (12.2%)	1.04	0.853	1.023 (0.670-1.561)	0.917
T	24 (6.9%)	83 (10.9%)	107 (9.6%)	72 (10.5%)	35 (8.2%)	1.32	0.203	1.213 (0.752-1.958)	0.428
U	40 (11.5%)	107 (14%)	147 (13.2%)	89 (13%)	58 (13.6%)	0.95	0.777	1.039 (0.691-1.563)	0.855
X	12 (3.4%)	27 (3.5%)	39 (3.5%)	27 (3.9%)	12 (2.8%)	1.42	0.319	1.348 (0.609-2.984)	0.461
Others (B,E,W,K,I)	15 (4.3%)	48 (6.3%)	63 (5.7%)	35 (5.1%)	13 (3%)	1.71	0.099	1.019 (0.557-1.865)	0.951

*Values after adjustment for age, sex, BMI and population. N, number of individuals; OR, odds ratio; CI, confidence intervals for OR as calculated using logistic regression model using PLIN.

**Table 3:** Sub-clade classification of individuals with mtDNA haplogroup H n> 1.

Sub-clade	Number of individuals
H	24
H14b	14
H2a	12
H6b	11
H13a	10
H13c	9
H14	9
H15a	5
H1	4
H29a	3
H2b	2
H4b	2
H5’36	2
H5c	2
H6	2
H6a	2

### mtDNA variants associated with T2D

The analysis identified 26 mtDNA variants that were nominally associated with T2D in the Arab Gulf region cohort, as presented in **Table 4**. These associations were determined after adjusting for key covariates including age, sex, mtDNA haplogroup, and population group. Of these 26 variants, 13 were found to be positively correlated with an increased risk of T2D, while the remaining 13 exhibited a negative correlation, suggesting a potential protective effect.

**Table 4:** mtDNA variants association of gulf region arabs for diabetes.

mtDNA variants	Gene	Consequence	Frequency in T2D	Frequency in Control	Frequency in H haplogroup	OR (95% CI)*	P-value*
MT:16186C>T	TP	upstream	0.019	0.026	0.024	0.292 (0.109-0.782)	0.014
MT:2352T>C	RNR2	non-coding	0.025	0.007	0.018	5.616 (1.381-22.84)	0.016
MT:4640C>A	ND2	missense	0.003	0.012	0.007	0.096 (0.013-0.683)	0.019
MT:14212T>C	ND6	synonymous	0.019	0.009	0.018	4.946 (1.27-19.26)	0.021
*MT:4991G>A*	*ND2*	*synonymous*	*0.020*	*0.033*	*0.025*	*0.362 (0.152-0.862)*	0.022
*MT:709G>A*	*RNR1*	*non-coding*	*0.142*	*0.172*	*0.154*	*0.638 (0.431-0.945)*	0.025
MT:8460A>G	ATP8	missense	0.019	0.005	0.018	6.762 (1.262-36.23)	0.026
*MT:8419T>C*	*ATP8*	*synonymous*	*0.001*	*0.002*	*0.001*	*0.04 (0.002-0.712)*	0.028
MT:9300G>A	CO3	missense	0.012	0.002	0.008	12.54 (1.296-121.4)	0.029
MT:10667T>C	ND4L	synonymous	0.010	0.002	0.007	14.21 (1.309-154.1)	0.029
*MT:12007G>A*	*ND4*	*synonymous*	*0.055*	*0.033*	*0.047*	*2.156 (1.079-4.305)*	0.030
MT:15670T>C	CYP	synonymous	0.016	0.002	0.011	13.81 (1.26-151.5)	0.032
MT:13101A>C	CYB	synonymous	0.009	0.002	0.006	13.7 (1.219-154.1)	0.034
MT:11204T>C	ND5	synonymous	0.006	0.014	0.009	0.225 (0.056-0.897)	0.035
MT:16304T>C	TP	upstream	0.046	0.019	0.038	2.537 (1.067-6.036)	0.035
MT:10816A>G	ND4	synonymous	0.009	0.002	0.006	13.34 (1.19-149.5)	0.036
MT:2000C>T	RNR2	non-coding	0.009	0.002	0.006	13.25 (1.182-148.5)	0.036
MT:13780A>G	ND5	missense	0.060	0.040	0.053	2.072 (1.044-4.113)	0.037
MT:9554G>A	CO3	synonymous	0.022	0.012	0.018	3.459 (1.069-11.19)	0.038
MT:4188A>G	ND1	synonymous	0.003	0.010	0.005	0.114 (0.014-0.916)	0.041
MT:13743T>C	ND5	synonymous	0.003	0.009	0.005	0.114 (0.014-0.917)	0.041
MT:9656T>C	CO3	synonymous	0.004	0.009	0.006	0.169 (0.031-0.935)	0.042
MT:8994G>A	ATP6	synonymous	0.007	0.016	0.011	0.282 (0.082-0.974)	0.045
MT:10685G>A	ND4L	synonymous	0.012	0.016	0.014	0.292 (0.087-0.975)	0.045
MT:14696A>G	TE	non-coding	0.001	0.002	0.002	0.053 (0.003-0.948)	0.046
*MT:16163A>G*	*TP*	*upstream*	*0.022*	*0.024*	*0.024*	*0.38 (0.145-0.998)*	*0.049*

mtDNA variants in italics were identified by conditional analysis as having a dependent effect on T2D with respect to the conditioned top SNP (MT:16186C>T) with the lowest p-value.

Among the positively associated variants, MT:2352T>C in the RNR2 gene stands out as a significant non-coding variant, with an odds ratio (OR) of 5.616 (95% CI = 1.381-22.84; p = 0.016), indicating a strong association with T2D. Similarly, the MT:8460A>G variant, a missense mutation in the ATP8 gene, was associated with T2D (OR = 6.762; 95% CI = 1.262-36.23; p = 0.026). This variant affects a key component of the mitochondrial ATP synthase complex, potentially leading to impaired ATP production and subsequent disruptions in insulin secretion and glucose metabolism.

Conversely, several variants were identified as having a protective association with T2D. Five of these variants, including MT:16186C>T, MT:4991G>A, MT:4188A>G, MT:709G>A, and MT:11204T>C, showed a significant negative correlation with T2D, indicating a protective effect. For example, MT:16186C>T, located in the non-coding D-loop region, exhibited a protective effect against T2D (OR = 0.292; 95% CI = 0.109-0.782; p = 0.014). This variant is likely involved in the regulation of mitochondrial DNA replication and transcription, possibly reducing the risk of T2D by improving mitochondrial function. Similarly, MT:4991G>A in the ND2 gene and MT:4188A>G in the ND1 gene, which are both involved in encoding subunits of NADH dehydrogenase, also demonstrated protective effects, suggesting that these variants might enhance oxidative phosphorylation efficiency, thereby mitigating T2D risk. MT:709G>A in the RNR1 gene and MT:11204T>C in the ND5 gene were additionally found to have protective associations, further emphasizing the role of mitochondrial variants in influencing metabolic health and disease resistance.

Further conditional analysis, focusing on the top leading variant MT:16186C>T, identified 20 additional variants that remained significantly associated with T2D, suggesting that these variants exert an independent effect on the risk of the disease. However, five variants from the initial 26 lost their significance after controlling for MT:16186C>T, indicating that their association with T2D was likely dependent on this leading variant. This dependency highlights the complex genetic interactions among mtDNA variants that contribute to T2D susceptibility.

In total, 514 mtDNA variants were identified exclusively in individuals with T2D, as detailed in **Supplementary Table S4**. **Table 5** highlights several significant variants that are uniquely present in the T2D group, including MT:15530T>C in the CYB gene and MT:6587C>T in the CO1 gene. These variants have been implicated in previous studies of metabolic disorders, further supporting their relevance in T2D within the Arab Gulf population.

**Table 5:** Significant associated mtDNA variants that present only in diabetic individuals.

mtDNA variants	Gene	Consequence	Number of individuals	P-value
MT:15530T>C	CYB	synonymous	8	0.027
MT:6587C>T	CO1	synonymous	7	0.048

## Discussion

In this study, we explored the association between mitochondrial haplogroups and T2D by combining data from Kuwaiti and Qatari cohorts, which individually might not have shown significant associations, possibly due to limited sample sizes. Given the environmental and genetic similarities of these populations (42), we combined the cohorts to increase the statistical power and offer a comprehensive view of the genetic landscape of Arabs in the Gulf region. The PCA analysis of mitochondrial haplogroups confirmed the ethnic homogeneity between the two populations. This integrated approach enhanced the reliability of our findings, enabling us to identify genetic markers and risk factors with greater confidence, thus providing valuable insights into the genetic factors associated with T2D.

In the current study, the mitochondrial H haplogroup was found to have a protective effect against obesity in the combined populations (OR/95% CI = 0.607/0.397-0.929; p = 0.021) after adjusting for age, sex, BMI, and population. Furthermore, the prevalence of individuals with the H haplogroup was higher in the control groups than in the T2D groups in both populations, although this difference was not statistically significant. Additional analysis revealed that most individuals with the H haplogroup belonged to the H14b and H2a subclades. Haplogroup H, although most prevalent in Europe, has a notable presence in the Arabian Peninsula. Studies have shown mean frequencies of around 9.4% in the region, with variations across different countries and regions (43). This distribution supports the relevance of our findings within the Gulf Arab populations. Furthermore, this haplogroup has been previously associated with a protective effect against obesity in Arabs living in Kuwait (44). Given that obesity is a significant risk factor for T2D, this protective effect might contribute to a reduced risk of T2D in individuals with haplogroup H. Furthermore, the H2a maternal lineage, common in the Saudi Arabian population, is associated with lean individuals in the Arab population living in Kuwait (44), likely contributing to the reduced T2D risk observed in the H2a subclade.

However, studies on Chinese Uyghur (45) and Bangladeshi populations (46) reported that haplogroup H was associated with an increased risk of T2D. This inconsistency with our results may be due to several factors. First, genetic background effects can differ significantly across populations, leading to inconsistent haplogroup associations. The different frequencies of H subclades across populations further complicate this, as our study identified specific subclades of haplogroup H, whereas these studies did not. Second, the Bangladeshi study only sequenced the D-loop hypervariable region I in their haplogroup classification (46). We previously observed a 71% concordance in mitochondrial haplogroup profiling between variants from the full mitochondrial genome extracted from whole-exome data and the D-loop region data from conventional Sanger sequencing. This suggests that using only the D-loop region could lead to haplogroup misclassification (47). Additionally, the small sample sizes in these studies may have contributed to the differing results. Another explanation involves observations of tightly coupled oxidative phosphorylation (OXPHOS) haplogroups, such as haplogroup H, which use fewer calories to produce more ATP. Another tightly coupled haplogroup has been associated with both elite athletes (48) and obesity (49, 50) in the Japanese population. Researchers hypothesize that the “thrifty genotype” could explain this phenomenon: while high ATP production efficiency benefits athletes, it predisposes individuals to obesity if they become sedentary later in life (48).

The prevalence of individuals with mitochondrial haplogroup T is higher in the T2D group compared to the control group in the Kuwaiti population, the Qatari population, and the combined population representing Arabs in the Gulf region. However, the difference in frequency was not significant. However, a significant association was observed for haplogroup T with an increased risk of T2D in the white Brazilian population (51). The T haplogroup was also associated with obesity in the southern Italian population (52) and in Austrian juveniles and adults (53).

mtDNA variants have been implicated as possible genetic causes of T2D. The T-to-C change at nucleotide position 16189, which creates a homopolymeric polycytosine (poly-C) tract between positions 16184 and 16193 within the D-loop region, has been linked to T2D in specific ethnic groups (20, 54, 55). This variant’s diabetogenic effect is thought to be connected to mtDNA replication issues and influenced (56) by factors such as increased body weight (57) and oxidative stress (58). However, the association has been inconsistent across different populations (23).

In our study involving Arabs from the Gulf region, we identified a significant protective variant: 16186 C>T (OR/95% CI = 0.292/0.109-0.782) after adjusting for covariates. This variant was the most significant in our study. Located within the same poly-C tract in the D-loop region, it replaces a cytosine with thymine, possibly impacting replication or regulation processes differently from the 16189 T>C variant, which was not prioritized in our study.

We found that the variant MT:2352T>C (OR/95% CI = 5.616/1.381-22.84) is positively correlated with T2D in our study. This variant was previously associated with decreased mitochondrial inner membrane potential during oxidative phosphorylation. The minor allele of this SNP is almost absent in individuals of Caucasian ancestry but is prevalent in those of African American ancestry, where it has been linked to an increased risk of fibromyalgia (59). Given its prevalence in the Arab population, its association with decreased cellular energy metabolism suggests a potential mechanism for its link to T2D through mitochondrial dysfunction and impaired insulin sensitivity (60).

Among the positively associated variants in our study, we identified a significant missense variant, MT:8460A>G, within the ATP8 gene. This gene encodes a subunit of ATP synthase in complex V, which utilizes the proton electrochemical gradient across the inner membrane during oxidative phosphorylation to synthesize ATP from adenosine diphosphate (61). Variants within the ATP8 gene have been shown to impact β-cell function and ROS generation in animal models (62) and have been associated with T2D in genetic studies (45).

Additionally, we identified a missense (MT:9300G>A) and a synonymous (MT:9554G>A) as significant variants in the cytochrome c oxidase subunit III (CO3) gene. A different variant in the CO3 gene has been linked to MIDD (63).

Variants in the Cytochrome B gene (CYB) have also been linked to MIDD (64) and T2D (39). In our study, we identified a significant synonymous variant (MT:13101A>C) in the CYB gene.

We found several significant variants positively correlated with T2D located in the nicotinamide adenine dinucleotide (NADH) dehydrogenase subunit genes (ND4, ND4L, and ND6) of respiratory complex I. NADH dehydrogenase is essential for energy generation; thus, variants within its encoding genes could result in metabolic disorders (65). Variants in these genes have been associated with MIDD (66) and T2D (67, 68). Similarly, we also found a significant synonymous variant in the RNR2 gene (MT:2000C>T), which encodes Humanin, a peptide shown to improve insulin sensitivity in animal models of diabetes mellitus (69). We also identified two significant synonymous variants that were found exclusively in the T2D groups: MT:15530T>C in the CYB gene and MT:6587C>T in the cytochrome c oxidase subunit I (COI) gene. A variant in the COI gene has been linked to MIDD (63).

Finally, we examined known mtDNA positions associated with T2D exclusively in individuals with T2D (70), and we identified five variants. Three of these are exact known variants from ND1 (37), CYB (39), and mitochondrially encoded tRNA glutamic acid (TE) (40) genes, all of which are associated with T2D. The other two variants share the same positions with known variants in literature but have different nucleotide changes. One is located within RNR2 at position MT:3200 (38) with a T>A change, and the other is within ND1 at position MT:3399 (41) with an A>G change (**Table 6**).

**Table 6:** Known diabetic associated mtDNA position that were in diabetic individuals only.

mtDNA variants	Gene	Frequency in T2D	Known variants	Diabetes	Population	Reference
MT:3357G>A	ND1	0.004	MT:3357G>A	T2D, T1D	Japan	(37)
MT:3200T>A	RNR2	0.001	MT:3200T>C	T2D	China	(38)
MT:15746A>G	CYB	0.004	MT:15746A>G	T2D	Taiwan	(39)
MT:14693A>G	TE	0.002	MT:14693A>G	T2D, MIDD	Taiwan	(40)
MT:3399A>G	ND1	0.001	MT:3399A>T	GDM*	Singapore	(41)

*GDM, Gestational diabetes mellitus.

This study acknowledges several limitations. First, the associations between mitochondrial haplogroups and T2D, as well as mitochondrial variants and T2D, did not reach statistical significance after applying Bonferroni correction, might indicate that the sample size is not large enough to accommodate multiple testing’s. Furthermore, diabetes is a multifactorial disorder influenced by a variety of factors. Including adjustments for lifestyle variables, dietary habits, and meal frequency could provide a more comprehensive analysis. Lastly, our results have not been validated in a larger, independent cohort of Arabs from the Gulf region, which is necessary to confirm these findings.

## Conclusion

In conclusion, this study provides important evidence that mitochondrial genetics play a significant role in modulating T2D risk in Arab populations of the Gulf region. The protective effect of haplogroup H, as well as the identification of specific disease-associated variants, highlight the value of investigating mitochondrial markers as part of a precision medicine approach to understanding and managing T2D in this high-risk population. Further research is warranted to elucidate the functional mechanisms underlying these genetic associations and to translate these findings into improved clinical screening and prevention strategies.

## Data Availability

The datasets presented in this study can be found in online repositories. The names of the repository/repositories and accession number(s) can be found in the article/**Supplementary Material**.
